# Supplementary material for: Maternal dysglycaemia, changes in the infant’s epigenome modified with a diet and physical activity intervention in pregnancy: Secondary analysis of a randomised control trial
Source: PLoS Med. 2020 Nov 5;17(11):e1003229. doi: 10.1371/journal.pmed.1003229 (PMC7643947; doi:10.1371/journal.pmed.1003229)
Supplement: S2 Text — (DOCX) [file pmed.1003229.s003.docx]

**Analysis Plan:**

1. **Purpose of proposed investigation**

The incidence of gestational diabetes mellitus (GDM) is increasing with the rising prevalence of maternal obesity. Children born to mothers with GDM have heightened risk of obesity and metabolic disease in adulthood, leading to an intergenerational cycle of metabolic disease. The purpose of this investigation is to improve understanding of the mechanisms through which GDM exposure *in utero* translates to the development of obesity in the children, and thereby inform preventative intervention strategies. Increasing evidence suggests that maternal GDM induces stable modifications of the offspring epigenome, resulting in persistently changed gene expression and a lifelong increased risk of obesity and associated disorders. Utilising the biobank of the largest randomised controlled trial (RCT) of a lifestyle intervention (low glycaemic index diet and increased physical activity) in obese pregnant women (UK Pregnancy Better Eating and Activity Trial, UPBEAT) ^(1)^, an RCT which lowered maternal dietary glycaemic index, increased physical activity^(1)^ and reduced the infant’s fat mass, this investigation will for the first time directly assess a) the role of epigenetic processes in mediating the effect of maternal dysglycaemia and GDM on offspring adiposity, b) the influence of fetal genotype, and c) how GDM treatment and maternal lifestyle behaviors can modify these epigenetic marks and offspring obesity risk. Thus, this study will provide unique insights into the mechanisms linking maternal dysglycaemia, GDM and lifestyle with childhood obesity, enabling the development of effective interventions to prevent or ameliorate this cycle of metabolic disease.

**Hypothesis:** We hypothesize that maternal GDM, gestational dysglycaemia and dietary glycaemic load in pregnancy act through epigenetic processes to increase the risk of obesity in the child, and that GDM treatment modality and maternal lifestyle behaviors modify this risk. Specifically, we will:

1. identify epigenetic changes in the fetal epigenome induced by maternal GDM, gestational dysglycaemia and dietary glycaemic load, the influence of the fetal genotype, and the pathways linking maternal biochemical drivers with epigenetic changes in the fetus.

2. determine whether a lifestyle intervention influences DNA methylation in the infant and/or modulates the methylation signature associated with maternal GDM/dysglycemia.

**Detailed Plan of Investigation**

**AIM 1:** ***Identify the epigenetic changes in the fetal epigenome induced by maternal GDM, gestational dysglycaemia and dietary glycaemic load; the influence of the fetal genotype and determine the pathways linking maternal biochemical drivers with epigenetic changes in the fetus.***

*Objective i):* Determine whether GDM (GDM n=179, controls n=465), gestational dysglycaemia (continuous variable) and dietary glycaemic load (continuous variable), are associated with differences in fetal methylation signatures using cord blood DNA and whether such changes are sex specific.

*Objective ii*) Determine the interaction of the fetal genotype with maternal GDM/gestational dysglycaemia/dietary glycaemia load on the fetal epigenotype to identify genotypic groups with differential sensitivity.

*Objective iii):* Identify gene networks/transcriptional hubs enriched amongst the differentially methylated CpGs (DMCpGs) and regions (DMRs) associated with maternal GDM/gestational dysglycaemia/dietary glycaemic load.

*Objective iv):* Determine which maternal biochemical markers linked to maternal GDM/gestational dysglycaemia/dietary glycaemia load (insulin, adiponectin, leptin, lipids, metabolome, inflammatory markers) in early (15-18^+6^ weeks) and late (27-28^+6^ weeks) gestation are associated with epigenetic changes in cord blood DNA.

*Objective v)*: Identify the mediators and pathways linking maternal GDM/gestational dysglycaemia/dietary glycaemia load with maternal biochemical markers to changes in the fetal epigenome using network discovery modelling.

***Analysis Plan for Aim1:***

***DNA methylation analysis****:* Genome-wide methylation will be assessed using the new Infinium Methylation EPIC BeadArray, which covers over 850,000 CpG sites, including >90% of the CpG sites on the original Illumina HumanMethylation 450K BeadArray together with CpGs sites located within DNase1 hypersensitive sites, ENCODE open chromatin, FANTOM5 enhancers, tissue-specific DMRs and miRNA promoters. The additional coverage over these regions is important as studies have shown that environmentally responsive CpG sites are located within open chromatin and enhancers. Thus, this approach will provide unique data for genomic regions not previously studied and valuable insights into the methylation changes associated with GDM.

*Data analysis:* The new EPIC array maintains the same chemistry and mixed probe design as the 450k array and therefore will enable the use of all previously designed robust packages ^(2)^ for QC, normalization and analysis. In addition to standard statistical approaches looking for differences in mean methylation at DMCpGs, we will also search for DMRs and VMRs as previously described ^(3; 4)^. We will adjust for the mixed cellular composition of whole blood^(3; 5)^ ; algorithms adapting this for cord blood have now been validated. Additionally, detailed blood subtype epigenomes via the BLUEPRINT consortium will enable external comparisons and validation ^(6)^. CpGs coinciding with SNPs, shown to cross hybridise to other genomic loci and on the sex chromosomes will be removed from the analysis

*Pyrosequencing:* Pyrosequencing will be used to validate the top ten DMCpGs/DMRs within the identified networks (Aims 1-4) in all 644 subjects.

*Identification of networks and pathways:* Potential enrichment of the differentially methylated genes of interest for particular gene ontology (GO) terms, biochemical (KEGG) pathways, disease association (OMIM) and protein interaction networks will be performed using ToppGene (oppgene.cchmc.org), MetaCore and GREAT^(7)^ . Functional exploration of DMRs will also be evaluated in ENCODE data via EpiExplorer (*Halachev K, Bast H, Albrecht F, Lengauer T, Bock C:****EpiExplorer: live exploration and global analysis of large epigenomic datasets.****Genome Biol (2012), 13:R96)* and transcription factor enrichment of DMRs via TRAP ^(8)^ and MEME-ChIP ^(9; 10)^ algorithms.

*Genotyping and causal analysis:* Samples from the RCT will be genotyped using the Illumina Infinium HumanOmniExpress Beadchip. The acquisition of high-resolution genetic data for all subjects will allow us to investigate the interaction of infant genotype with maternal GDM, dysglycaemia and dietary glycaemic load. The existence of methylation quantitative trait loci (meQTL) is widespread throughout the human genome and across multiple tissues ^(11; 12; 13)^ and sequence changes can modulate methylation levels *in cis* ^(14)^. ^(15; 16; 17)^.. For the DMRs identified in Aim1 we will determine if there are *in cis* polymorphisms which form meQTLs with the DMRs. Further, we will determine if the polymorphisms interact with maternal GDM, dysglycaemia and dietary glycaemic load to affect fetal methylation differentially across genotypic groups.

***Statistical Analysis***: Analyses will be undertaken in Stata and R as appropriate. We will assess the relationships of DNA methylation values (transformed where necessary) with maternal GDM, gestational dysglycaemia & dietary glycaemic load, with secondary analyses of the maternal markers listed previously, here we will consider UPBEAT a cohort study, including the intervention as a covariate in analyses and including interactions as appropriate. Where necessary, distributions of methylation values will be transformed to an approximate Normal distribution and t-tests for independent samples used to examine differences in mean methylation; if not possible, non-parametric (e.g. Mann-Whitney U) tests will be used to examine group differences (e.g. median). Multivariable linear regression (with variable transformation/categorisation and exploration of non-linear relationships as appropriate) will be used to assess the effect of gestational dysglycaemia, dietary glycaemic load and maternal biochemical markers on cord blood CpG methylation. Gender, intervention group and appropriate covariates will be included in these models. Residuals will be checked to ensure that assumptions for linear regression have been met. For each multivariable regression analysis, potential covariates (including cellular composition (Houseman method, as in our recent studies), maternal age, ethnicity, parity, smoking pre- and in pregnancy, diet and physical activity at baseline and socioeconomic position) will be decided a priori based on the existing literature and mechanistic knowledge. To check that maternal exposures, have a similar effect in the intervention and control groups, maternal exposure X group interactions will be examined. Differential network analysis, principal components analysis, and multivariable models will be used to explore the relationship and overlap between epigenetic changes associated with maternal GDM/dysglycaemia/dietary glycaemic load and maternal biochemical markers.

**AIM 2**: ***Determine whether the lifestyle intervention that lowers maternal dietary glycaemic index and increases physical activity influences the epigenetic marks associated with maternal GDM /dysglycemia.***

*Objective i)*: Evaluate the effect of the lifestyle intervention (n=324 Control, n=320 Intervention) on DNA methylation in the infant.

*Objective ii):* Evaluate the effect of intervention on the GDM and dysglycemia association dmCpgs

**Analysis plan for Aim 2:**

DNA methylation analysis: The influence of intervention on DNA methylation in the infant will be assessed as described above. Briefly, limma in R will be used to run multivariate linear regressions to determine the associations between DNA methylation and the intervention after adjustment for pre-determined covariates. The dmCpGs/DMRs and networks associated with intervention and the potential influence of common genotype on these will also be examined and changes induced by GDM, and intervention compared by principal component and differential network analyses. In addition to assess whether the intervention may modulate the GDM and dysglycemia associated dmCpGs, intervention will be added as a co variate in the main GDM and dysglcyemia EWAS analysis. If differences found after adjustment, then the association of DNA methylation with GDM/dysglycemia will be analysed separately in the two arms of the cohort and effect sizes compared. Data from the two groups (intervention/standard care) or mode of treatment will be analysed either on an ‘intention to treat’ or ‘as treated’ basis as appropriate, with other statistical approaches as under Aim 1.

**Sample size and power calculations**:

Our primary analysis will involve a total of 644 samples with i) 179 GDM cases and 465 controls and ii) 324 Intervention and 320 standard care. The array contains 868,565 probes, so a Bonferroni correction of standard p<0.05 criteria would be 5.8E-8. However, DNA methylation at individual CpGs has been shown to correlated over short chromosomal distances ^(18)^. Grouping contiguous CpGs into comethylated regions will add power and reduce false-positive results. Using the method outlined in Ong and Holbrook ^(3)^, we estimated that the 483,000 probes on the Infinium450K array would reduce to 55,003 regions to be tested against phenotype. Although the EPIC array is less sparse and irregular in coverage than the 450K if we extrapolate from the 450K, the EPIC will have approximately 98,910 co-methylated regions, yielding an alpha of 5.06E-7 . Alternatively using the Storey false discovery rate correction, a less conservative multiple testing procedure, ^(19)^ which may be more appropriate given the interdependence of the regions and used in Finer et al ^(20)^, the alpha will be higher(5.06E-6). Although , effect size is unknown, Finer et al ^(20)^, found that “top hit MVPs” which passed FDR for GDM exposed (n=27) or not (n=21) in cord blood, differed in absolute methylation between 5.3 to 14.9%. Standard deviation at these loci are not readily available in the paper but assuming 10%, the effect size is approximately 0.5-1.49. Using these calculations at 644 sample size for our primary analyses of GDM and Intervention we have 90% power to detect a 0.55 effect size at likely alpha value 5.06E-6^(21)^. In a secondary analyses the effect of treatment on the fetal epigenome will be examined, here numbers per group are lower although effect size may be larger so this will be carried out as a discovery screen, followed by pathway analyses to identify significantly enriched networks. For both the primary and secondary analyses we will seek to replicate these findings in publicly available data sets and in the Southampton Women Survey (SWS) and GUSTO cohorts, 2 mother-offspring cohorts.


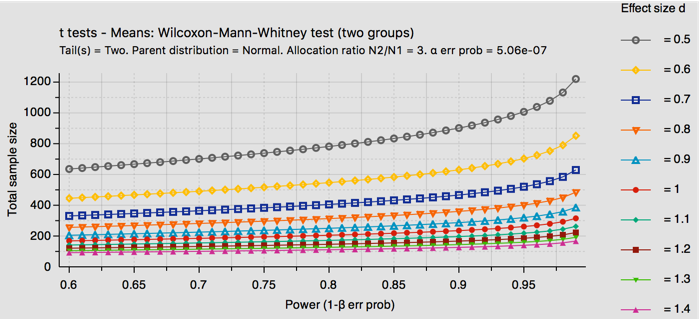


Figure 1 shows that with a sample size of 644 (and performing two-tailed non-parametric Mann-Whitney tests) there shall be 90% power to detect an effect size of >0.6. There will be >99% power to detect an effect size of >0.7 and 60% power to detect an effect size of 0.5.

.

1. Poston L, Bell R, Croker H *et al.* (2015) Effect of a behavioural intervention in obese pregnant women (the UPBEAT study): a multicentre, randomised controlled trial. *Lancet Diabetes Endocrinol* **3**, 767-777.

2. Pan H, Chen L, Dogra S *et al.* (2012) Measuring the methylome in clinical samples: improved processing of the Infinium Human Methylation450 BeadChip Array. *Epigenetics : official journal of the DNA Methylation Society* **7**, 1173-1187.

3. Ong ML, Holbrook JD (2014) Novel region discovery method for Infinium 450K DNA methylation data reveals changes associated with aging in muscle and neuronal pathways. *Aging cell* **13**, 142-155.

4. Jaffe AE, Murakami P, Lee H *et al.* (2012) Bump hunting to identify differentially methylated regions in epigenetic epidemiology studies. *IntJEpidemiol* **41**, 200-209.

5. Houseman EA, Ince TA (2014) Normal cell-type epigenetics and breast cancer classification: a case study of cell mixture-adjusted analysis of DNA methylation data from tumors. *Cancer informatics* **13**, 53-64.

6. Adams D, Altucci L, Antonarakis SE *et al.* (2012) BLUEPRINT to decode the epigenetic signature written in blood. *Nature biotechnology* **30**, 224-226.

7. McLean CY, Bristor D, Hiller M *et al.* (2010) GREAT improves functional interpretation of cis-regulatory regions. *Nature biotechnology* **28**, 495-501.

8. Thomas-Chollier M, Hufton A, Heinig M *et al.* (2011) Transcription factor binding predictions using TRAP for the analysis of ChIP-seq data and regulatory SNPs. *Nature protocols* **6**, 1860-1869.

9. Machanick P, Bailey TL (2011) MEME-ChIP: motif analysis of large DNA datasets. *Bioinformatics* **27**, 1696-1697.

10. Ronn T, Volkov P, Gillberg L *et al.* (2015) Impact of age, BMI and HbA1c levels on the genome-wide DNA methylation and mRNA expression patterns in human adipose tissue and identification of epigenetic biomarkers in blood. *Human molecular genetics* **24**, 3792-3813.

11. Gibbs JR, van der Brug MP, Hernandez DG *et al.* (2010) Abundant quantitative trait loci exist for DNA methylation and gene expression in human brain. *PLoS genetics* **6**, e1000952.

12. Bell JT, Pai AA, Pickrell JK *et al.* (2011) DNA methylation patterns associate with genetic and gene expression variation in HapMap cell lines. *Genome biology* **12**, R10.

13. Zhang D, Cheng L, Badner JA *et al.* (2010) Genetic control of individual differences in gene-specific methylation in human brain. *American journal of human genetics* **86**, 411-419.

14. Lienert F, Wirbelauer C, Som I *et al.* (2011) Identification of genetic elements that autonomously determine DNA methylation states. *Nature genetics* **43**, 1091-1097.

15. Liu Y, Aryee MJ, Padyukov L *et al.* (2013) Epigenome-wide association data implicate DNA methylation as an intermediary of genetic risk in rheumatoid arthritis. *Nat Biotechnol* **31**, 142-147.

16. Liu Y, Li X, Aryee MJ *et al.* (2014) GeMes, clusters of DNA methylation under genetic control, can inform genetic and epigenetic analysis of disease. *American journal of human genetics* **94**, 485-495.

17. Kato N, Loh M, Takeuchi F *et al.* (2015) Trans-ancestry genome-wide association study identifies 12 genetic loci influencing blood pressure and implicates a role for DNA methylation. *Nature genetics* **47**, 1282-1293.

18. Eckhardt F, Lewin J, Cortese R *et al.* (2006) DNA methylation profiling of human chromosomes 6, 20 and 22. *Nature genetics* **38**, 1378-1385.

19. Storey JD (2002) A direct approach to false discovery rates. *Journal of the Royal Statistical Society, Series B* **64**, 479-498.

20. Finer S, Mathews C, Lowe R *et al.* (2015) Maternal gestational diabetes is associated with genome-wide DNA methylation variation in placenta and cord blood of exposed offspring. *Human molecular genetics* **24**, 3021-3029.

21. Faul F, Erdfelder E, Lang AG *et al.* (2007) G*Power 3: a flexible statistical power analysis program for the social, behavioral, and biomedical sciences. *Behav Res Methods* **39**, 175-191.
