# Supplementary figures and images for: Maternal dysglycaemia, changes in the infant’s epigenome modified with a diet and physical activity intervention in pregnancy: Secondary analysis of a randomised control trial
Source: PLoS Med. 2020 Nov 5;17(11):e1003229. doi: 10.1371/journal.pmed.1003229 (PMC7643947; doi:10.1371/journal.pmed.1003229)

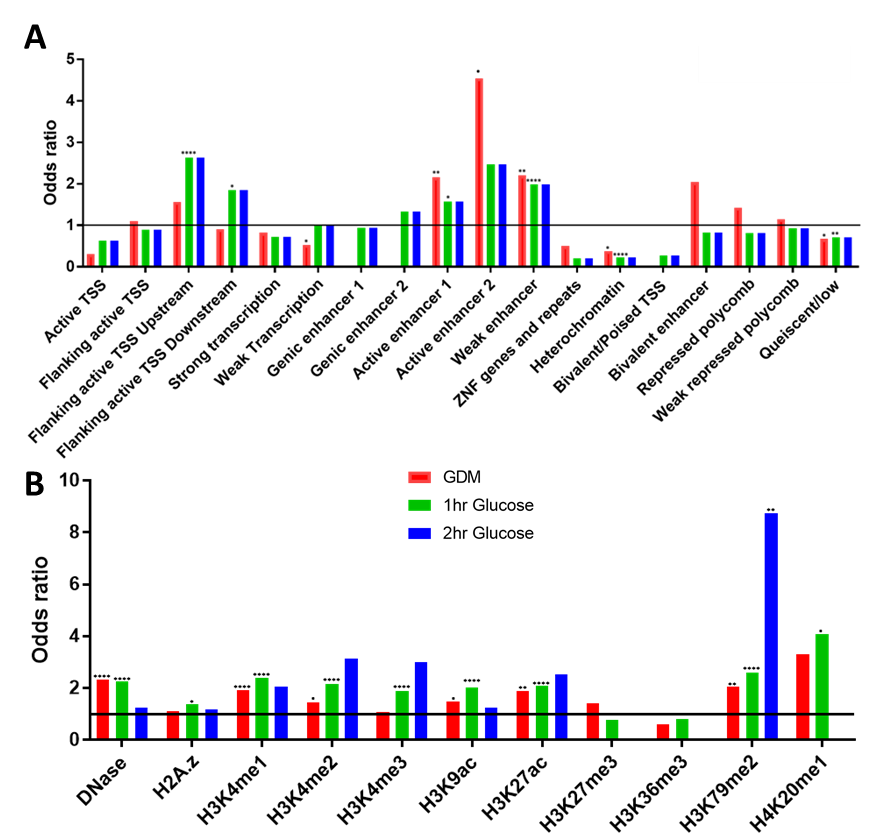

Supplement: S1 Fig — (A) Enrichment of dmCpG among different chromatin states as determined by the ENCODE Hidden Markov Model in HUVECs. (B) Overlap of dmCpGs with different histone modifications (H). Enrichment was calculated using the Fisher exact test. *p < 0.05, **p < 0.01, ***p < 0.001, ****p < 0.0001. (TIF) [file pmed.1003229.s004.tif]

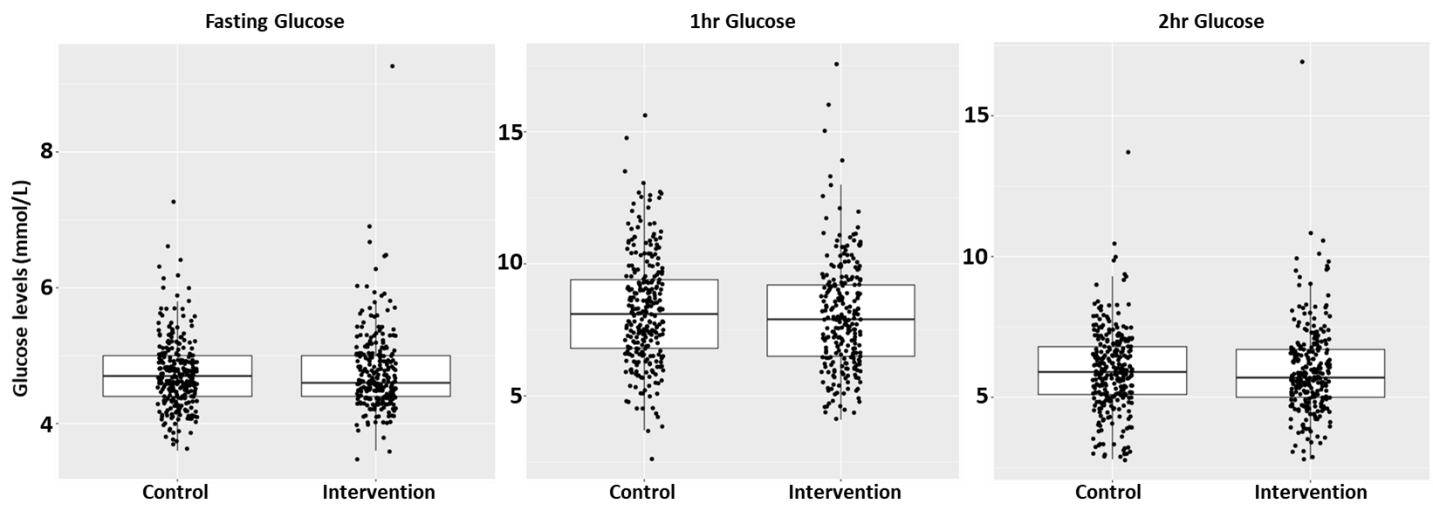

Supplement: S2 Fig — Comparison of (A) fasting glucose, (B) 1-h glucose, and (C) 2-h glucose levels separated by intervention arm, showing no difference in either measure between the 2 groups. (TIF) [file pmed.1003229.s005.tif]
