# Supplementary material for: Maternal dysglycaemia, changes in the infant’s epigenome modified with a diet and physical activity intervention in pregnancy: Secondary analysis of a randomised control trial
Source: PLoS Med. 2020 Nov 5;17(11):e1003229. doi: 10.1371/journal.pmed.1003229 (PMC7643947; doi:10.1371/journal.pmed.1003229)
Supplement: Attachment — Submitted filename: Review of Antoun at al.docx [file pmed.1003229.s006.docx]

**Review of Maternal Dysglycemia Induces Changes in the Infant’s Epigenome Which Are Ameliorated by a Diet and Physical Activity Intervention in Pregnancy**

The current study investigated the impacts of maternal GDM and continuous glucose levels on cord blood DNA methylation and additionally examined how lifestyle interventions that improve maternal glycemic control influence these relationships. Major strengths of the study include the design, sample size, and the acquisition of genetic data, which allowed the authors to evaluate how genetic variation influences their findings. However, there are also some important limitations. In particular, I do not believe that the statistical analyses performed were appropriate for testing the authors’ hypothesis that the lifestyle treatments modify the impacts of GDM/glucose on cord blood methylation. Several key references were also missing from the Introduction and Discussion. I also have concerns about the methods used to conduct the pathway analyses. Some additional minor concerns are also described in more detail below.

Introduction:

Line 73: There is a typo in the first sentence of the Introduction

Lines 95-96: Although most studies on GDM/glucose and methylation have been small, the PACE consortium recently published a meta-analysis on GDM and cord blood methylation, which merits some brief discussion in the Introduction (PMID: 31601636).

Lines 96-98: This statement is not true: “all have focussed on GDM versus no GDM, rather than the continuous relationship between maternal glucose levels and DNA methylation”. Some previous studies have evaluated maternal glucose continuously in relation to offspring methylation (e.g., see PMID: 29752424 and 22396200). There is also a typo in this sentence.

Methods:

Lines 188-189: “CpGs known to cross-hybridise to other locations in the genome (n=14,759), coinciding with SNPs (n=77,261)”. Please cite the appropriate supporting reference here.

Lines 214-218: Please provide some detail about how covariates were selected

Were results similar after additional adjustment for gestational age at OGTT? This needs to be addressed.

Network analyses: Do these analyses account for the fact that some genes are overrepresented on the EPIC array? There are software and methods available that account for this that may be more appropriate

Results:

Were any of the CpGs still statistically significant after applying the more conservative Bonferroni correction? This is worth reporting.

Did the DMRs identified using DMRcate overlap with the differentially methylated CpGs identified using the traditional EWAS single CpG approach? Or were there notable differences? It would be interesting to see more details on this.

Lines 389-391: “The increase in number of dmCpGs associated particularly with 1h-PG levels after adjustment for intervention suggested that the intervention might attenuate the 1h-PG associated methylation signature in the infant”. This rationale isn’t clear to me. Wouldn’t you expect to see fewer differentially methylated CpGs after adjusting for treatment? Also, if effect modification is of interest, the main comparison should be the results stratified by treatment arm (i.e., Table 2). However, rather than conducting an EWAS for these stratified analyses, it would make more sense to identify CpGs that are significantly differentially methylated by glucose level in the control group and then compare the magnitudes of the effect estimates for these same loci in the intervention arms.

Discussion:

Lines 421-422 “There is increasing evidence that maternal dysglycemia has adverse effects on the health of the offspring, predisposing the infant to developing obesity and metabolic disease in later life.” Please provide supporting references for this statement.

Lines 461-462 “higher maternal 1h-PG levels concentrations reflect impaired insulin secretion”. Please provide supporting references for this statement.

Additional limitations:

Please be explicit about the lack of gene expression data

Please address generalizability as a limitation

Please address the lack of follow-up data on methylation in childhood as a limitation (or a potential future direction)

Lines 519-521: “More recently, a meta-analysis of seven pregnancy cohorts (3,677 mother-newborn pairs with 317 GDM cases) identified 2 DMRs associated with GDM within OR2L13 and CYP2E1”. The citation for this study is missing.
