# Supplementary material for: Maternal dysglycaemia, changes in the infant’s epigenome modified with a diet and physical activity intervention in pregnancy: Secondary analysis of a randomised control trial
Source: PLoS Med. 2020 Nov 5;17(11):e1003229. doi: 10.1371/journal.pmed.1003229 (PMC7643947; doi:10.1371/journal.pmed.1003229)
Supplement: Attachment — Submitted filename: Rebuttal final ea (3).docx [file pmed.1003229.s007.docx]

**Editors comments:**

**1.Your title must be nondeclarative and not a question. It should begin with main concept if possible. "Effect of" should be used only if causality can be inferred, i.e., for an RCT. Please place the study design ("A randomized controlled trial," "A retrospective study," "A modelling study," etc.) in the subtitle (ie, after a colon).**
We have now amended the title accordingly to: **Maternal dysglycaemia, changes in the infant’s epigenome and amelioration by a diet and physical activity intervention in pregnancy: secondary analysis of a randomised control trial**

**2. .Abstract
* Please include the study design, population and setting, number of participants, years during which the study took place, length of follow up, and main outcome measures.**

*** Please quantify the main results (with 95% CIs and p values).**

*** Please include the important dependent variables that are adjusted for in the analyses.**

*** In the last sentence of the Abstract Methods and Findings section, please describe the main limitation(s) of the study's methodology.**
We have now amended the abstract accordingly to include more detail on the study design, participants, dependent variables, main results and limitations.

**3.Abstract Conclusions:
* Please address the study implications without overreaching what can be concluded from the data; the phrase "In this study, we observed ..." may be useful.
* Please interpret the study based on the results presented in the abstract, emphasizing what is new without overstating your conclusions.**
The conclusion has now been amended. The amended conclusion now states ‘Maternal dysglycaemia was associated with significant changes in the epigenome of the infants. Moreover, we found that the epigenetic impact of a dysglycaemic prenatal maternal environment was modified by a lifestyle intervention in pregnancy.

**4.Author summary
At this stage, we ask that you include a short, non-technical Author Summary of your research to make findings accessible to a wide audience that includes both scientists and non-scientists. The Author Summary should immediately follow the Abstract in your revised manuscript. This text is subject to editorial change and should be distinct from the scientific abstract. Please see our author guidelines for more information:** [**https://eur03.safelinks.protection.outlook.com/?url=https%3A%2F%2Fjournals.plos.org%2Fplosmedicine%2Fs%2Frevising-your-manuscript%23loc-author-summary&amp;data=01%7C01%7CK.A.Lillycrop%40soton.ac.uk%7C43c700fadc3245d9eb0a08d7fb3ce8c2%7C4a5378f929f44d3ebe89669d03ada9d8%7C0&amp;sdata=IbngeBdQfagtdEVJvFyuDztLqlU%2BZmvqywsEJhp69Ic%3D&amp;reserved=0**](https://eur03.safelinks.protection.outlook.com/?url=https%3A%2F%2Fjournals.plos.org%2Fplosmedicine%2Fs%2Frevising-your-manuscript%23loc-author-summary&amp;data=01%7C01%7CK.A.Lillycrop%40soton.ac.uk%7C43c700fadc3245d9eb0a08d7fb3ce8c2%7C4a5378f929f44d3ebe89669d03ada9d8%7C0&amp;sdata=IbngeBdQfagtdEVJvFyuDztLqlU%2BZmvqywsEJhp69Ic%3D&amp;reserved=0)
An author summary has now been included.

**5.References should be placed within square brackets please and bibliography should be in Vancouver style**

All references have been changed to square brackets and Vancouver style.

**6.Methods
Please mention where the original trial took place**

This has now been included in the methods (lines 173-175).

**7.Did your study have a prospective protocol or analysis plan? Please state this (either way) early in the Methods section.**
The original analysis plan has been added as a supplementary information file, S1 Text

**a) If a prospective analysis plan (from your funding proposal, IRB or other ethics committee submission, study protocol, or other planning document written before analyzing the data) was used in designing the study, please include the relevant prospectively written document with your revised manuscript as a Supporting Information file to be published alongside your study, and cite it in the Methods section. A legend for this file should be included at the end of your manuscript.
b) If no such document exists, please make sure that the Methods section transparently describes when analyses were planned, and when/why any data-driven changes to analyses took place.
c) In either case, changes in the analysis-- including those made in response to peer review comments-- should be identified as such in the Methods section of the paper, with rationale.**
The analysis plan from the original funding proposal has been included as a Supporting Information file and cited in the Methods section (lines 309-310) with a legend for the file at the end of manuscript.

**8.Discussion
Lines 428-432 need to be toned down. Please add “to our knowledge” to avoid assertions of primacy. In addition, since this study can be strictly described as “observational” I would avoid the use of causal language.**
This sentence has been re written as advised, and we have avoided the use of causal language.

**9.Please ensure that the study is reported according to the STROBE guideline and include the completed checklist as Supporting Information. When completing the checklist, please use section and paragraph numbers, rather than page numbers. Please add the following statement, or similar, to the Methods: "This study is reported as per the Strengthening the Reporting of Observational Studies in Epidemiology (STROBE) guideline (S1 Checklist)."**

The STROBE check list is now included, and we have added the following sentence to the Methods section – ‘This study is reported as per the Strengthening the Reporting of Observational Studies in Epidemiology (STROBE) guideline (SI Checklist)."

**Reviewer #1: This manuscript capitalises on samples and date generated from women who participated in UPBEAT (a) to address the relationship between maternal glycaemia and cord blood DNA methylation patterns and (b) to examine the ability of a lifestyle intervention (diet and physical activity) to modify these associations. The study has several strengths (including the large numbers, validation of the array data and consideration of heterogeneity) but I have several comments that the authors should consider:

1. The women included in the current study represent about a third of those in the total UPBEAT study. How did the characteristics of the subset differ from the whole cohort and on what basis were they selected (or what determined if a buffy coat from cord blood was collected)?**
We have now added a table to the supplementary information where we have compared the characteristics of the whole cohort with the subset of samples analyzed in this study. The cohort characteristics were similar, albeit with some differences in maternal age, parity and ethnicity which are now described in the Results section. We have also explained in the Results section that the subset of samples analyzed here were the only samples for which buffy coat was available (lines 321-324, table S2).

**2. It is mentioned that fetal sex was included in the regression models. However, given that there is accumulating evidence for the importance of sex difference in the field, it is surprising that sex specific effects are neither investigated nor discussed.**

Because of the growing evidence of sex differences in the field, we have as the reviewer suggested analysed the effect of maternal GDM/dysglycaemia on male and female infants separately. This analysis showed that at an FDR<0.05, there were 4 GDM associated dmCpGs in male infants and 0 in females, while for 1hr glucose, there are 75 dmCpGs associated with 1hr-PG in males and 112 dmCpGs in females; importantly there was no overlap between the 1hr-PG associated dmCpGs in the male and female infants. This analysis has now been added into the Results section of the paper with the implications of the findings discussed (lines 402-410, 626-635).

**3. It is a strength that validation of the array data by pyrosequencing was included. What were the % validation rates observed?**
The % validation rates were 100%. Of the 3 assays run on the pyrosequencer, all showed the same results as the array, including the 3 extra CpGs in the BRD2 assay. We have made this clearer in the text (lines 504-505).

**4. Was any threshold for methylation differences incorporated in the analysis? Some differences look to be very small - discussion of the potential biological relevance of difference is important.**
In the analysis, probes with an absolute methylation range < 10% were removed from the dataset to select the more variable probes to carry out the analysis on. The subsequent changes observed in the EWAS analysis were still relatively small, although in keeping with previous studies investigating the effect of maternal GDM or adverse early life environments on DNA methylation in the infant. As suggested, we have now included a discussion of the potential functional importance of such small methylation changes (lines 697-702).

**5. How many of the loci as being identified as being differentially methylated relate to genes that are expressed in cord blood cells? Given the relatively late nature of the intervention, many of its effects on methylation are likely to be tissue specific, therefore discussion of potential functional relevance is important.**
At present there isn’t a gene expression data base for cord blood on the GTEx Portal. There is for whole blood samples, but cord blood has a very different composition to peripheral blood so a comparison may be of limited value. As the reviewer states given the late nature of the intervention, many of the effects on methylation may be tissue specific; we have included a discussion of the potential functional significance of the changes in DNA methylation and the limitation that we did not have RNA from the same samples to assess whether the methylation changes were accompanied by a change in expression (lines 703-708).

**6. Line 471- the statement that high 1 hour glucose reflects impaired insulin secretion needs a reference.**
We have now included the following references to support this statement: Florentino et al (2015), Paddock et al (2017) and Marini et al (2012).

**7. Line 547 - the term widespread is probably not appropriate - it is actually a small percentage of loci affected**
This has been corrected.

**8. Table 1 - glucose and insulin measurements are probably not accurate to two decimal places.**

This has been corrected.

**Reviewer #2: Thanks for the opportunity to review your manuscript. My role is as a statistical reviewer, so I have concentrated my review around the data and analysis (and the presentation of these). I should declare that I do not have bioinformatics expertise and I recommend that someone with expertise in this area review the manuscript. I have included general comments and queries to begin with, followed by questions that are more specific.

This study is a secondary analysis of data from a randomised control trial comparing usual care with a diet and physical activity intervention in pregnant women with a BMI >30. The main study found that the intervention was successful in improving diet and physical activity, but there were no differences detected in the primary outcomes of GD development and LGA infants. In this secondary study the authors have examined whether DNA methylation levels from infants were associated with maternal GD status (and other continuous glycaemic measures)

This is an interesting study and there are some improvements I think could be made to the current manuscript.**

**1.As PLOS Medicine has a general audience, it would be helpful for an explanation of the key outcomes (i.e. the measurements of methylation) to be included so that the importance of this work is obvious to someone without extensive experience in genomics.**
We have now added to the discussion an explanation of the key outcomes and discussed how DNA methylation changes may impact gene transcription and consequently cell function and metabolism. We have also sought to clarify the analyses we have carried out for a non-specialist audience by adding further detail to the Methods and Results sections. In addition, we have added an author summary.

**2.Was a protocol or analysis plan created for this secondary study? If this were available, it would be helpful in reviewing.**
An analysis plan which was created for this study is now included as a Supplementary Information file. (S1 Text)

**3.How was missing data accounted for in the analysis? The rates of missingness are not extremely high, but there are key measurements (fasting insulin, HBA1c etc.) with a reasonable level of missing data and my impression from a look at the tables is that this may depend on GD diagnosis. Was a complete-case analysis used or a listwise deletion strategy?**
Missing data was handled on a case by case basis, using a listwise deletion strategy rather than a complete-case analysis. We have added information on missingness and how it was accounted for in the Methods section on statistical analysis on lines 299-300. For fasting insulin and HBA1c, where there was a greater degree of missingness, these measurements weren’t used in the analysis and are therefore removed from table 1.

**4.L193. 'Hierarchical clustering' is vague, what exact algorithm and metrics were used for this analysis?**

Euclidean distance was calculated for the samples, and hierarchical clustering using complete linkage as implemented in the hclust function in R was used to cluster the samples. Further detail on this has now been added into the Methods on lines 238-240.

**5.L214. What form did the covariates take in the regression model (continuous, polynomial, or as dummy variables)?**

Age and cell composition estimates were all continuous and smoking, ethnicity, parity and sex were included as dichotomous variables. This information is now included in the Methods section on lines 262-275.

**6.How were assumptions (linearity, heteroscedacity, distribution of residuals) checked?**
For the full EWAS, it wasn’t possible to check these assumptions for the ~300,000 regressions that were carried out. However, to account for the regression diagnostics, we checked the QQ plots, heteroscedasticity, Cook’s distance and the residuals of the top 5 dmCpGs obtained in each analysis. All further regression analysis, including the pyrosequencing validation had the regression diagnostics checked to ensure assumptions are met. These further details have been added to the text in the Method section on lines 307-309

**7.L218. More details are needed about the sensitivity analysis - how exactly were these conducted? Are these sub-group analyses or a type of moderation/mediation model?**

For the sensitivity analyses of maternal BMI, gestational age at OGTT and intervention, these were added individually into the models as covariates. Further detail on these analyses are now described in the Methods section (Lines 267-270)

**8.L218. FDR is a much more utilised concept for controlling for multiplicity in genomic analysis as opposed to clinical/epidemiology where we are much more used to familywise methods. I do not think the application of the B-H method here is inappropriate but it does achieve power at the cost of higher Type I rates. Is FDR generally accepted as appropriately for looking at how patient level measurements are associated with levels of methylation?**
B-H is the generally accepted method to correct for multiple testing when looking at measures associated with DNA methylation and is standard method for these arrays. We did also carry out Bonferroni adjustment which is now reported on line 336-337, where 7 CpGs associated with GDM passed this threshold. However, Bonferroni also has its downsides because it is a more stringent form of multiple testing correction and can introduce more false negatives into the results.

**9.L253/Figure S1. Fisher's exact test can be used where all the margins of the tables are fixed, which I don't think is the case with this data. There are alternatives e.g. Barnard's test for when 1 or more margins aren't fixed.**
We considered using Barnard’s but for these large table sizes the power advantage has been reported to be minimal ( Mehta, CR 1993 [*doi*](https://en.wikipedia.org/wiki/Doi_(identifier))*:*[*10.1080/00031305.1993.10475946*](https://doi.org/10.1080%2F00031305.1993.10475946)) and the computational time very long.

**10.L355. Is there are way to present point estimates/effect size measures from these linear models in addition to just the p-values?**

R squared values as a measure of effect size are now included in the text, see lines 479-483 and 503-504.

**11.Table 1. The summary information here is useful but I don't think that the p-values are useful, given that a null hypothesis of no difference isn't realistic given the groups are stratified according to GDM diagnosis. I would just remove the p-values.**

The p-value column has been removed.

**Reviewer 3:**

**Review of Maternal Dysglycemia Induces Changes in the Infant’s Epigenome Which Are Ameliorated by a Diet and Physical Activity Intervention in Pregnancy**

**The current study investigated the impacts of maternal GDM and continuous glucose levels on cord blood DNA methylation and additionally examined how lifestyle interventions that improve maternal glycemic control influence these relationships. Major strengths of the study include the design, sample size, and the acquisition of genetic data, which allowed the authors to evaluate how genetic variation influences their findings. However, there are also some important limitations. In particular, I do not believe that the statistical analyses performed were appropriate for testing the authors’ hypothesis that the lifestyle treatments modify the impacts of GDM/glucose on cord blood methylation. Several key references were also missing from the Introduction and Discussion. I also have concerns about the methods used to conduct the pathway analyses. Some additional minor concerns are also described in more detail below.**

**Main points:**

**Is the statistical analysis performed appropriate for testing whether the lifestyle intervention modifies the impact of GDM on cord blood DNA methylation?**

The Reviewer suggested to address this question we should in the stratified analysis also compare the effect sizes of the dmCpGs. We have now as suggested by the Reviewer included this further analysis - Please see Point (10+11) where we have covered this in more detail. But briefly comparison of the effect sizes of the GDM/dysglycaemic associated dmCpGs in the intervention and standard care arm of the trial, showed that the effect sizes of 87.8% of the GDM and 77.5% of the 1hr-PG dmCpGs are reduced in the intervention arm compared to the control arm, supporting a modifying effect of the intervention on the dysglycaemia signature in the infant.

**References**

Further references have been included, and references moved from the Discussion to the Introduction as suggested. Please see below for more details.

**Pathway analysis:**

We had included in the manuscript a pathway analysis which didn’t account for the bias of the number of CpGs per gene. We have now repeated the pathway analysis using methylglm which accounts for such bias. This has identified similar pathways. This analysis is now included in the manuscript. Please see Point 7 for further details.

Introduction:

**1)Line 73: There is a typo in the first sentence of the Introduction**

This has been corrected.

**2)Lines 95-96: Although most studies on GDM/glucose and methylation have been small, the PACE consortium recently published a meta-analysis on GDM and cord blood methylation, which merits some brief discussion in the Introduction (PMID: 31601636).**

We had included a discussion of the PACE consortium GDM analysis in the discussion, but we have now also described their findings in the introduction. Please see lines 140-142.

**3) Lines 96-98: This statement is not true: “all have focussed on GDM versus no GDM, rather than the continuous relationship between maternal glucose levels and DNA methylation”. Some previous studies have evaluated maternal glucose continuously in relation to offspring methylation (e.g., see PMID: 29752424 and 22396200). There is also a typo in this sentence.**

We have revised this sentence as it wasn’t clear that this sentence referred to cord blood EWAS. We have now included references to the studies by Cardenas and Bouchard which looked at maternal glucose levels with respect to placental DNA methylation. See lines 142-144. We have also amended the typo in the sentence.

Methods:

**4.Lines 188-189: “CpGs known to cross-hybridise to other locations in the genome (n=14,759), coinciding with SNPs (n=77,261)”. Please cite the appropriate supporting reference here.**

We have now added in the reference; please see line 234.

**5.Lines 214-218: Please provide some detail about how covariates were selected**.

We used the Directed Acyclic Graph (DAG) approach to select the co-variates; covariates known to affect DNA methylation in the child (maternal smoking, neonate sex, ethnicity, maternal age, cell composition) as well as those affecting the risk of GDM (maternal age, ethnicity) were identified by DAG. This approach has now been described in the Methods section (lines 260-265).

**6.Were results similar after additional adjustment for gestational age at OGTT? This needs to be addressed.**

As suggested by the reviewer, we have now analyzed the data with an additional adjustment for gestational age at OGTT. Here, we found considerable overlap between the maternal GDM/dysglycaemia associated dmCpGs in the gestational age adjusted and unadjusted analysis. There was an 86% overlap of the dmCpGs significantly associated with GDM after adjustment for gestational age at OGTT with the GDM associated dmCpGs identified in the initial analysis. Furthermore, there was significant overlap between the 1h-PG -dmCpGs (58%), 2h-PG -dmCpGs (82%) and FPG-dmCpG (100%) and the dmCpGs after adjustment of the regression models for gestational age at OGTT. These results are now described in the Results section. (lines 346-349 and 399-401).

**7.Network analyses: Do these analyses account for the fact that some genes are overrepresented on the EPIC array? There are software and methods available that account for this that may be more appropriate**

The STRING analysis was used to identified enriched networks of genes differentially methylated but does not account for the fact that some genes are overrepresented on the array. Nonetheless it does provide a way of connecting the genes with dmCpGs to determine whether there are functional clusters of genes with significant dmCpGs. We have as requested by the reviewer now carried out a second pathway analysis using the *methylglm* function from the methylGSA package, which accounts for the bias of number of CpGs per gene. This has identified similar pathways. We have now added this to the Pathway section (lines 279-281 and 451-456).

**Results:**

**8.Were any of the CpGs still statistically significant after applying the more conservative Bonferroni correction? This is worth reporting.**

Maternal GDM was associated (false discovery rate corrected p<0.05) with altered methylation status of 242 CpG loci in cord blood of infants, of which 7 dmCpGs remained significant after the more conservative Bonferroni adjustment for multiple testing. This data has been added to the Results section (lines 336-337).

**9.Did the DMRs identified using DMRcate overlap with the differentially methylated CpGs identified using the traditional EWAS single CpG approach? Or were there notable differences? It would be interesting to see more details on this**.

There was overlap between the DMRs and the dmCpGs associated with GDM. Of the 47 GDM-associated DMRs, 20 of the regions also included significant GDM associated dmCpGs. This information is now described in the Results section (lines 463-464 and Table S15).

**10.Lines 389-391: “The increase in number of dmCpGs associated particularly with 1h-PG levels after adjustment for intervention suggested that the intervention might attenuate the 1h-PG associated methylation signature in the infant”. This rationale isn’t clear to me. Wouldn’t you expect to see fewer differentially methylated CpGs after adjusting for treatment?**

After we adjusted for intervention in the main analysis, we saw an increase in the number of CpG associated with 1h-PG levels. The increase in the number of dmCpGs potentially reflects an improvement in the model after accounting for the intervention, suggesting that the intervention might modify the 1hr-PG associated methylation signature in the infant. But attenuate as the Reviewer points out is the wrong word to use to describe this, so as suggested by the reviewer we have re-written this section to explain why after observing the increase in the number of CpGs associated with 1h-PG levels we then carried out a stratified analysis. (lines 523-526)

**11.Also, if effect modification is of interest, the main comparison should be the results stratified by treatment arm (i.e., Table 2). However, rather than conducting an EWAS for these stratified analyses, it would make more sense to identify CpGs that are significantly differentially methylated by glucose level in the control group and then compare the magnitudes of the effect estimates for these same loci in the intervention arms.**

There were two aims of the study. The first aim was to determine the effect of maternal GDM/dysglycaemia on cord blood DNA methylation of infants born to obese pregnant mothers, a high risk under-studied population. The second aim was to assess the impact of the lifestyle intervention on the GDM/dysglycaemia associated methylation signature in the infants. So, in table 2 we reported the results of these two analyses.

To gain further insights into the effect of the intervention on the glycemia associated methylation signatures, we carried out an EWAS stratified by intervention and found differences between the two arms. Furthermore, as suggested by the reviewer we have now compared the magnitudes of the effect estimates for the same loci in the control and intervention arm. Comparison of the effect sizes for the 41 dmCpGs associated with GDM in the control arm of the study with their effect size in the intervention arm showed that for 87.8% of these dmCpGs, there was a reduction in the effect size in the intervention arm compared to the control arm of the study. Similarly, comparison of the 160 dmCpGs associated with 1h-PG in the control arm, with the intervention arm showed that 77.5% of the effect sizes were reduced in the intervention arm for these dmCpG sites. For example, cg03566881(LGR6) and cg16536918 (AVP), two of the top hits associated with GDM, showed a decrease in effect size of 0.0772 and 0.0332 respectively in the intervention arm compared to the standard care arm, with similar decreases in the 1hr-PG analysis.

This is now reported in the Results section at lines 538-547, as well as table S21 to show the results.

Discussion:

**12.Lines 421-422 “There is increasing evidence that maternal dysglycemia has adverse effects on the health of the offspring, predisposing the infant to developing obesity and metabolic disease in later life.” Please provide supporting references for this statement.**

We have now added the following references: Lowe et al (2019), Scholtens et al (2019) and Gomes et al (2018).

**13.Lines 461-462 “higher maternal 1h-PG levels concentrations reflect impaired insulin secretion”. Please provide supporting references for this statement.**

We have added the following references which support this statement: Florentino et al (2015), Paddock et al (2017) and Marini et al (2012).

**14.Additional limitations: Please be explicit about the lack of gene expression data**

There was no RNA from the cord blood DNA samples to allow for a comparison with methylation status – this is now discussed as a limitation on lines 706-708.

**15.Please address generalizability as a limitation.**

We have now addressed the generalizability of our findings in the limitations and discussed the need for further studies to address the optimum gestational window for intervention and replication of findings in other pregnancy intervention trials where lifestyle interventions have been used to improve glycemic control in both lean and obese women (lines 719-723).

**16.Please address the lack of follow-up data on methylation in childhood as a limitation (or a potential future direction).**

We have added a section on the need to follow up the children in terms of methylation and health outcomes to determine whether the reduced dysglycaemic methylation imprint after intervention is accompanied by improve health outcomes in the children (lines 731-733).

**17.Lines 519-521: “More recently, a meta-analysis of seven pregnancy cohorts (3,677 mother-newborn pairs with 317 GDM cases) identified 2 DMRs associated with GDM within OR2L13 and CYP2E1”. The citation for this study is missing.**

This citation has been added.
