# Supplementary material for: Maternal dysglycaemia, changes in the infant’s epigenome modified with a diet and physical activity intervention in pregnancy: Secondary analysis of a randomised control trial
Source: PLoS Med. 2020 Nov 5;17(11):e1003229. doi: 10.1371/journal.pmed.1003229 (PMC7643947; doi:10.1371/journal.pmed.1003229)
Supplement: Attachment — Submitted filename: corrections 29th sept.docx [file pmed.1003229.s008.docx]

Corrections

**1.We still require confirmation of authorship and a declaration of any potential competing interests in relation to this manuscript from co-authors Michael S Kobor and Julie MacIssac. We have re-sent our authorship email, but also request that you contact these co-authors directly to ensure they respond.**
We have contacted the authors and they are expecting an email from PLOSMed

**2. Please indicate the corresponding author on the title page with an asterisk (*) and include the corresponding author's email address. Only an asterisk and the email address itself are required.

Please format as follows, omitting all other information:

*** [**kal@soton.ac.uk**](mailto:kal@soton.ac.uk)

This has been amended

**3. Please ensure that all Supporting Information files use the following format verbatim throughout the manuscript (including legends and all in-text citations): S1 Fig, S1 Table, S1 Text, S1 Data, etc. Each file type should start at 1 and increase numerically. Please note, however, that the file names themselves must use an underscore rather than a space, i.e. S1_Fig.tif, S1_Table.xlsx, S1_Text.doc, S1_Data.xlsx, S1_PRISMA_Checklist.docx, etc.**
The filenames have been amended accordingly

**4. Please note that your figures do not currently comply with our publication requirements. For further instructions to assist with your Figure revisions, please refer to our Figure Guidelines at:** [**https://eur03.safelinks.protection.outlook.com/?url=http%3A%2F%2Fjournals.plos.org%2Fplosmedicine%2Fs%2Ffigures&amp;data=01%7C01%7CK.A.Lillycrop%40soton.ac.uk%7C14d36fd00239479fb50308d85fd90390%7C4a5378f929f44d3ebe89669d03ada9d8%7C0&amp;sdata=qiw53ladenOruWa8QcqI2YON2HOLqcyIhe5Cvoh%2Fjfw%3D&amp;reserved=0**](https://eur03.safelinks.protection.outlook.com/?url=http%3A%2F%2Fjournals.plos.org%2Fplosmedicine%2Fs%2Ffigures&data=01%7C01%7Ck.a.lillycrop%40soton.ac.uk%7C37b5c9ed6a6642fbb70208d8620b1bf2%7C4a5378f929f44d3ebe89669d03ada9d8%7C0&sdata=rjoFPjw97r2GlTEaeinWnlC%2BXxNFMaKVwmf%2F7gt1OVM%3D&reserved=0)**.

If you need assistance, please contact us directly at** [**graphics@jjeditorial.com**](mailto:graphics@jjeditorial.com) **so we can further help with this matter. Please send any relevant source files and tell us what application(s) and what operating system you are using.

-Figures 1-5 have text that looks smaller than 8 points. The legibility of type at sizes smaller than 8 points in the published PDF is frequently compromised. We cannot assume responsibility for the legibility of Figures in the published PDF if** **there is type smaller than 8 point in them. Please note that this problem will not be detected or corrected by the PACE tool. We recommend that you print figures out to get a better idea of the text legibility. If you are happy with the way they look, we will publish them with the font as is. We would recommend using the max width of 19.05cm and height of 22.25cm to give yourself more space to work with.**
We have amended the figures and enlarged the text where possible. Some of the lettering still remains font 8 but enlarging it further would mean the text overlaps. We are happy with the figures as they now are and believe they confer the necessary formation

-**Text in Figures 2 and 3 look like they were originally at a lower resolution and will appear blurry or pixelated if published. Text and images should appear crisp and clear. Please note that this problem will not be detected or corrected by the PACE tool, and this will not be fixed by simply increasing the resolution of your Figures in the image editing software. Please carefully review the PLoS Figure Guidelines and provide Figure files that were originally created at 300 pixels/inch (dpi) and at least 6.68cm wide, but less than 19.05cm and under 22.225cm tall. For further information, please see the Figure Guidelines (**[**https://eur03.safelinks.protection.outlook.com/?url=http%3A%2F%2Fjournals.plos.org%2Fplosmedicine%2Fs%2Ffigures&amp;data=01%7C01%7CK.A.Lillycrop%40soton.ac.uk%7C14d36fd00239479fb50308d85fd90390%7C4a5378f929f44d3ebe89669d03ada9d8%7C0&amp;sdata=qiw53ladenOruWa8QcqI2YON2HOLqcyIhe5Cvoh%2Fjfw%3D&amp;reserved=0**](https://eur03.safelinks.protection.outlook.com/?url=http%3A%2F%2Fjournals.plos.org%2Fplosmedicine%2Fs%2Ffigures&data=01%7C01%7Ck.a.lillycrop%40soton.ac.uk%7C37b5c9ed6a6642fbb70208d8620b1bf2%7C4a5378f929f44d3ebe89669d03ada9d8%7C0&sdata=rjoFPjw97r2GlTEaeinWnlC%2BXxNFMaKVwmf%2F7gt1OVM%3D&reserved=0)**).**
We have amended the text so that the image is clearer.
